# Modelling the immunosuppressive effect of liver SBRT by simulating the dose to circulating lymphocytes: an in-silico planning study

**DOI:** 10.1186/s13014-018-0952-y

**Published:** 2018-01-22

**Authors:** L. Basler, N. Andratschke, S. Ehrbar, M. Guckenberger, S. Tanadini-Lang

**Affiliations:** University Hospital Zurich, Department of Radiation Oncology, University of Zurich, Rämistrasse 100, CH 8091 Zürich, Switzerland

**Keywords:** Immunotherapy, Modelling, Treatment planning, Stereotactic body radiotherapy (SBRT), Abscopal effect

## Abstract

**Background:**

Tumor immune-evasion and associated failure of immunotherapy can potentially be overcome by radiotherapy, which however also has detrimental effects on tumor-infiltrating and circulating lymphocytes (CL). We therefore established a model to simulate the radiation-dose delivered to CL.

**Methods:**

A MATLAB-model was established to quantify the CL-dose during SBRT of liver metastases by considering the factors: hepatic blood-flow, −velocity and transition-time of individual hepatic segments, as well as probability-based recirculation. The effects of intra-hepatic tumor-location and size, fractionation and treatment planning parameters (VMAT, 3DCRT, photon-energy, dose-rate and beam-on-time) were analyzed. A threshold dose ≥0.5Gy was considered inactivating CL and CL0.5 (%) is the proportion of inactivated CL.

**Results:**

Mean liver dose was mostly influenced by treatment-modality, whereas CL0.5 was mostly influenced by beam-on-time. 3DCRT and VMAT (10MV-FFF) resulted in lowest CL0.5 values of 16 and 19%. Metastasis location influenced CL0.5, with a mean of 19% for both apical and basal and 31% for the central location. PTV-volume significantly increased CL0.5 from 27 to 67% (10MV-FFF) and from 31 to 98% (6MV-FFF) for PTV-volumes ranging from 14cm^3^ to 268cm^3^.

**Conclusion:**

A simulation-model was established, quantifying the strong effects of treatment-technique, tumor-location and tumor-volume on dose to CL with potential implications for immune-optimized treatment-planning in the future.

**Electronic supplementary material:**

The online version of this article (doi: 10.1186/s13014-018-0952-y) contains supplementary material, which is available to authorized users.

## Background

Despite continuous multidisciplinary efforts, the prospect to transform advanced tumors into a state of “chronic disease” are still limited. Immunotherapy has shown encouraging clinical results by enhancing or inducing tumor-specific immune responses [1], however, tumor immune evasion represents a major challenge of cancer treatment today [2–4].

Major reasons for immune evasion are the immunosuppressive microenvironment of the tumor and insufficient infiltration of immune competent cells into the tumor [1, 5–11]. Radiotherapy has been demonstrated to overcome the immunosuppressive tumor microenvironment [12] and anecdotal reports suggest that local tumor irradiation may also exert systemic or abscopal anti-tumor effects by immune-response modification with subsequent response of non-irradiated tumor metastases [13–22]. Radiation does however also have detrimental effects not only on tumor infiltrating lymphocytes but also on circulating lymphocytes (CL) in the bloodstream during radiotherapy, as these cells are particularly radiosensitive [23]. In addition, prolonged lymphopenia during or after the tumor treatment has been shown to be a prognostic factor for overall survival in many cancer types and radiotherapy may be an important factor [24–27]. In this regard, circulating lymphocytes should be treated as a radiosensitive organ at risk.

Conventional fractionated radiotherapy could have an increased negative impact compared to hypofractionated approaches such as SBRT, because of usually larger irradiated volumes and overall treatment times of several weeks. This might lead to a significantly larger percentage of lymphocytes receiving a dose of more than 0.5 Gy, which is considered as a threshold dose for impaired lymphocyte function [23, 28]. The use of stereotactic body radiotherapy (SBRT) has increased rapidly in recent years and changed the field of radiation therapy in general, as well as outcome for select patients tremendously [29, 30]. Nearly all studies combining radiotherapy and immunotherapy focus on the use of SBRT with high ablative doses and a low number of fractions or even single fraction stereotactic radiosurgery (SRS).

This study aimed to establish a model to quantify the radiation dose delivered to CL. The proportion of circulating lymphocytes exposed to at least 0.5 Gy (CL0.5) was used as a surrogate parameter for radiation-induced immunosuppression. The model was established for SBRT of intra-hepatic metastases because many cancer types metastasize into the liver and liver SBRT is well established in the radiation oncology community. We analyzed the influence of various SBRT planning and delivery parameters such as dose rate, beam energy, treatment time, fractionation and treatment technique on the immunosuppressive effects of radiotherapy; additionally, the influence of PTV volume and intra-hepatic tumor location were investigated.

## Methods

### Model setting

For this in-silico planning study, three virtual liver metastases, positioned at different intrahepatic locations, were planned with SBRT of 3 × 15 Gy.

**Fig. 1:**
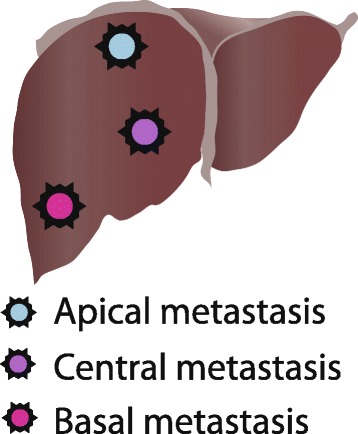
Intrahepatic locations of the three virtual liver metastasis

A contrast-enhanced CT scan of the liver was used for treatment planning. Contouring was performed in the treatment planning software Eclipse (Varian Medical Systems). Contours included the whole liver (1195 cm^3^), the hepatic segmentation into eight individual segments (I – VIII), portal vein, hepatic artery, inferior vena cava, aorta, lungs, spleen and kidneys. Contouring was performed according to the normal organ contouring guidelines for Radiation Therapy Oncology Group (RTOG) trials [31]. Three virtual liver metastases with a spherical diameter of 2 cm were delineated with either apical (a), basal (b) or central (c) location (Fig. 1). A PTV margin of 0.5 cm was added leading to a spherical diameter of 3 cm and a PTV volume of 14 cm^3^. While we kept the lesion size equal for all tumor locations and treatment modalities for comparability, we generated three additional larger lesions in the central location to evaluate volume-effects on circulating lymphocytes. Larger GTV diameters of 4, 5 or 7 cm were chosen resulting in PTV volumes of 65 cm^3^, 113 cm^3^ and 268 cm^3^, respectively.

Eclipse was used for treatment planning and DVH dose calculation. A fractionation of 3 × 15 Gy was chosen with an inhomogeneous dose prescription to the PTV encompassing 75% isodose line and treatment plans were normalized to a mean GTV dose of 56.25 Gy in all plans and treatment modalities. In total, 58 plans were generated using 3D conformal radiotherapy (3DCRT) and volumetric modulated arc therapy (VMAT). Details of planning characteristics are described in Table 1. To evaluate potential fractionation effects, two additional VMAT and 3DCRT fractionation schemes were analyzed (10 × 4.5 Gy and 20 × 2.25 Gy), normalized to the same mean GTV dose of 56.25 Gy.

**Table 1:** Total beam-on time (BOT), mean liver dose (MLD) and proportion of circulating lymphocytes receiving ≥0.5Gy (CL0.5) by treatment modality, beam energy and dose rate as a mean for all intrahepatic locations

1. Treatment modalities and mean MLD and CL0.5 of all locations							
Treatment modality	Beam energy	Flattening filter	Dose rate (MU/min)	Fields / Arcs	Total BOT	MLD	CL0.5
3DCRT	10 MV	FFF	2400	7	57 s	4.3 Gy	15.9%
3DCRT	6 MV	FFF	1400	7	111 s	4.3 Gy	23.6%
3DCRT	6 MV	FF	600	7	253 s	4.3 Gy	30.9%
VMAT	10 MV	FFF	2400	2	74 s	3.6 Gy	19.0%
VMAT	6 MV	FFF	1400	3	147 s	3.6 Gy	23.6%
VMAT	6 MV	FF	600	4	363 s	3.6 Gy	25.1%

### Model description

Cumulative dose to CLs was calculated using a liver segment specific DVH based convolution algorithm with the following assumptions:

1. About 7–9% of the arterial and 20–23% of the intestinal bloodflow pass through the liver, which equals to 27–32% of the total cardiac output [32, 33]. We used a value of 30% for our model.
2. Regional hepatic blood flow is comparable in the different liver segments [34].
3. Mean hepatic blood flow velocity is 10 mm/s.
4. Total body blood volume is 5 l.
5. Cardiac output is 5 l/min, resulting in a circulation time of 60 s for the total blood volume.
6. Mean hepatic transition time is different in each segment and based on proximity to arterial blood supply and venous drainage. It was estimated using the distance to the geometric center of each liver segment from the arterial (hepatic artery) and venous (portal vein) blood supply and venous drainage (hepatic veins). Hepatic transition time per segment varied from 7 to 23 s (see Additional file 1).
7. Same “volumes” of the blood stream were considered to be able to reenter the treatment field between different arcs/beams and treatment fractions, as the gantry rotation to the next arc or beam takes longer than the average hepatic transition and heart-to-heart circulation time.
8. A dose of 0.5 Gy and greater was considered effective in inactivating or killing circulating lymphocytes. This dose cutoff has been chosen by previous groups working on modelling CL irradiation and is based on the intrinsic radiosensitivity of the different lymphocyte subsets [23].
9. The probability of reentering a specific liver segment and thus treatment field, was calculated based on the percentage of cardiac output and relative volumes of the segments to generate a probability-based DVH convolution algorithm. As the cardiac output to the liver was defined as 30%, the probability of reentering during each cycle the liver itself was 30%, while the probability of reentering a specific segment varied from 2 to 22% (see Additional file 2).

We implemented an in-house developed model in MATLAB (Mathworks) to estimate the dose delivered to circulating lymphocytes based on the assumptions above.

### Statistical analysis

The statistical analysis was performed in MATLAB, graphs and illustrations were generated in GraphPad Prism and Adobe Illustrator. The analysis considered absolute and relative values of all treatment modalities for each individual metastasis location, as well as mean values of CL0.5, CL1.0, CL2.0, MLD, dose rate, number of fractions and beam-on time.

## Results

**Fig. 2:**
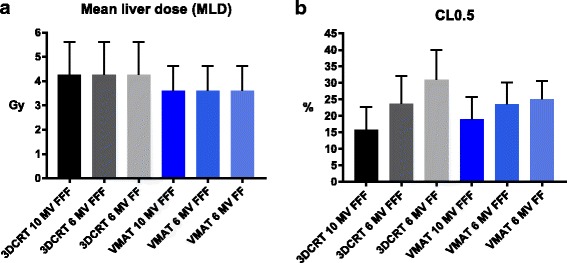
Mean liver dose (MLD, **a**) and proportion of circulating lymphocytes receiving ≥0.5Gy (CL0.5) by treatment modality as mean values with SD for all intrahepatic locations (**b**)

Mean liver dose (MLD) was mostly influenced by treatment modality: lowest values were achieved by VMAT followed by 3DCRT (Fig. 2a). In contrast, the proportion of CLs receiving ≥0.5Gy (CL0.5) was mostly influenced by beam-on time (BOT), which is associated with beam energy and dose rate. Mean BOTs of 3DCRT were 57 s (10MV FFF), 111 s (6MV FFF) and 253 s (6MV FF), while total VMAT BOTs were 74 s (10MV FFF), 147 s (6MV FFF) and 363 s (6MV FF).

Seven-field 3DCRT using 10MV FFF beams followed by VMAT (10MV FFF) resulted in lowest mean CL0.5 values of 16 and 19% (Fig. 2b). Treatment techniques using 6MV or 6MV FFF beam energy, which resulted in longer BOTs, showed substantially higher CL0.5 values with a mean of 24% or 31% for the 3DCRT and 24% or 25% for the VMAT plans, respectively (Table 1). Additionally, metastasis location influenced CL0.5, with a mean of 19% (11–25%) for the apical, 19% (13–27%) for the basal and 31% (24–41%) for the central location (Table 2).

**Table 2:** Mean liver dose (MLD) and proportion of circulating lymphocytes receiving ≥0.5Gy (CL0.5) by intrahepatic locations as a mean for all treatment modalities and beam energies

2. Dependence on tumor location		
Tumor location	MLD	CL0.5
Apical	3.1 Gy	18.8%
Basal	3.4 Gy	19.0%
Central	5.3 Gy	31.3%

**Fig. 3:**
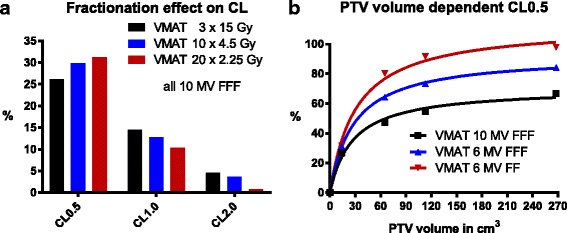
Effect of either 3 × 15 Gy, 10 × 4.5 Gy or 20 × 2.25 Gy fractionation on CLs (**a**) with VMAT 10MV FFF as treatment modality. 3DCRT did not show a relevant fractionation effect (see Additional file 4). **b.** PTV volume dependent CL0.5 by treatment modality, based on either 14 cm^3^, 65 cm^3^, 114 cm^3^ or 268 cm^3^ PTV sizes in the central tumor metastasis

We observed only a small fractionation effect for the VMAT technique (Fig. 3a). For 10MV FFF beam (central location) CL0.5 increased from 27% (3 fractions) over 30% (10 fractions) to 32% (20 fractions). We did not observe a fractionation effect for 3DCRT (see Additional file 3). An increase in PTV volume in the central tumor location led to a rapid increase of CL0.5 for all treatment modalities. Once the volume reached about 100 cm^3^, this influence reached a plateau and differences between modalities scaled linearly with a further increase in PTV volume (Fig. 3b). CL0.5 of VMAT (6MV FF) increased from 31% (14 cm^3^) to 80% (65 cm^3^), 91% (113 cm^3^) and 98% (268 cm^3^), while CL0.5 of VMAT (10MV FFF) only increased from 27% (14 cm^3^) to 47% (65 cm^3^), 55% (113 cm^3^) and 67% (268 cm^3^), respectively.

**Fig. 4:**
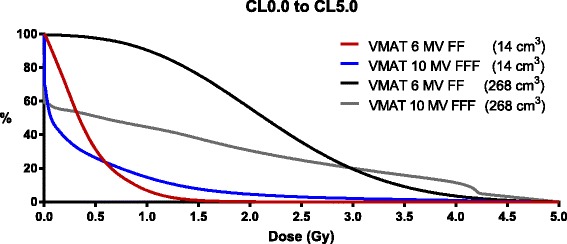
CL0.0 to CL5.0 for VMAT 10MV FFF and 6MV FF in the central tumor location and either 14 cm^3^ or 268 cm^3^ PTV volume

We also calculated the proportion of CLs receiving ≥1Gy (CL1.0), as well as CLs receiving ≥2.0Gy (CL2.0). Higher dose rates with shorter BOTs led to increased CL1.0 and CL2.0 values, while decreasing overall CL0.5 values and general low dose exposure of CLs (Fig. 4). In larger PTV volumes, this effect was not present anymore and higher dose rates decreased overall dose to CLs, including CL0.5, CL1.0 and CL2.0 with only a slight increase of doses greater than 3 Gy.

## Discussion

Identifying predictive biomarkers for the response to immunotherapy is challenging and complex. Currently only the expression of PD-L1 is widely used clinically but there are several limitations and for many malignancies PD-L1 expression alone might be insufficient for patient selection [35, 36]. Peripheral blood markers have been available for a long time and as previously stated: lymphopenia has been proposed as a prognostic factor for overall survival in many cancer types [24–27] and increased lymphocyte counts have also been associated with increased response and survival in the setting of immunotherapy [37–40]. It is also important to note that tumor-associated antigens released by immunogenic cell death of tumor cells are being generated during the time of radiation and have a short half-life. The time-frame around this antigen release might be most important for the induction of a systemic immune response. Consequently, decreasing the detrimental effects of radiotherapy on circulating lymphocytes might lead to a further benefit in these scenarios.

We hypothesized that volume and location of the treated liver metastasis as well as mean liver dose would be relevant factors influencing the radiation dose delivered to circulating lymphocytes. Another assumption was that volumetric arc therapy (e.g. VMAT) with its increased spread of low dose has increased immunosuppressive effects and 3D–CRT might have advantages with decreased low dose areas and shorter beam-on times at the cost of decreased high-dose conformity.

We confirmed most of our assumptions but observed that dose rate and treatment time had a stronger impact on CL0.5 than MLD, which was mostly influenced by treatment-modality. FFF beams in both 3DCRT and VMAT drastically reduced beam-on-time resulting in significantly lower CL0.5 values compared to lower beam energies (e.g. 6MV-FF).

The Johns Hopkins and Memorial Sloan-Kettering groups have shown that SBRT had a smaller effect than conventional radiotherapy (CRT) on the total lymphocyte count (TLC) in patients with unresectable pancreatic cancer [41]. In general, patients with a higher post-treatment TLC also showed a longer survival. The Oregon Clinic group showed similar results with neoadjuvant chemoradiation for borderline resectable and locally advanced pancreatic cancer. SBRT with 30 Gy in 3 fractions over 1 week and concurrent Gemcitabine minimized treatment-associated lymphopenia and reduced systemic loss of T cells, which was common in patients receiving conventional fractionated radiotherapy (CRT) with 50.4 Gy in 28 fractions over 5.5 weeks [42]. Additionally, the majority of patients receiving CRT failed to normalize their TLC post-treatment and it took up to 2 years in cases where TLC normalization occurred. Most of the SBRT patients however where able to normalize their TLC within the treatment period.

The Johns Hopkins group showed that the total dose to circulating lymphocytes increases rapidly with the number of fractions [23]. This effect is most likely based on assumption (8) of lymphocyte re-entry, which was also part of their analysis and considers possible reentering (sub-fractions) of the cells for every field (3D–CRT) or arc (VMAT) into the treatment field. In our model, we could not see a fractionation effect with 3DCRT and noticed only a minor fractionation effect in the VMAT. Differences to previous models may be the result of our probability-based approach of each individual liver segment and implementation of differing hepatic transition times compared to the single OAR approach of Yovino et al. In contrast to the results of Yovino et al., BOT and dose rate seem to be very important factors. We used higher dose rates with up to 2400 MU/min without flattening filter, which might explain some of the differences. With higher dose rates, less circulating lymphocytes are irradiated and a smaller portion of lymphocytes receives a higher dose, as shown in Fig. 4.

In the studies of Wild and Crocenzi [41, 42], PTV size was significantly larger for the conventionally fractionated radiotherapy (89 cm^3^ SBRT vs. 345 cm^3^ CRT in the first and approximately 150cm^3^ vs. 400cm^3^ in the second study). Thus, we built a model based on equal PTV volumes, while trying to optimize intra-fractional lymphocyte sparing by choice of treatment modality, dose rate and treatment time. PTV volume showed substantially larger differences than fractionation in our analysis, suggesting that the effect described in the studies above might be volume rather than fractionation related, though this would have to be confirmed in future in-vivo experiments.

PTV volume and treatment time seem to be the most critical factors of lymphocyte sparing. In summary, immunosuppressive effects of SBRT might be minimized by avoiding treatment of large metastases, a high number of fractions and especially long treatment times in the setting of immunotherapy.

There are limitations to our model, including that the value of CL0.5 is based on lymphocyte radio-sensitivity which has been assessed in vitro via colony formation assay [28] and in vivo data on dose-dependent activity and function of circulating lymphocytes is scarce [43–46]. As seen in Fig. 4, we are however able to minimize not only CL0.5 but also overall radiation exposure of CLs with the right treatment modality. Additionally, radiation sensitivity of lymphocytes is highly dependent on the specific subset. Our model is of most relevance to naïve T-cells, which are especially radiosensitive, compared to other subsets such as memory cells [47–49]. In addition, overall survival may be associated with a certain lymphocyte subset [50] and also be dependent on other cell types, e.g. innate immune cells such as neutrophils as discussed by Son et al. [51]. Regeneration and redistribution of lymphocytes, as well as local/non-circulating lymphocytes were not considered in our model and might influence the total amount of irradiated lymphocytes. This could also be dependent on tumor location and entity. However, this would likely affect all treatment modalities and inclusion of these factors would lead to a very complex model for which several parameters are unknown. Additionally, we do not know about the real impact of circulating lymphocytes on the efficacy of immunotherapy and immune-modulating antibodies may have additional influences on lymphocytes, including alteration and differentiation of subsets, as well as radiation sensitivity. However, the dependency of increased survival on absolute lymphocyte count suggests that we may be able to reduce detrimental radiation effects in this regard. The development of models for clinical decision-making is necessary for personalized treatment strategies in the metastatic situation [52]. These models should, however, be based on multiple biological and physiological parameters validated in-vivo prior to clinical application [53].

## Conclusion

A simulation-model has been established to estimate and quantify potential immunosuppressive effects of radiotherapy through inactivation of circulating lymphocytes. From a technical perspective, treatment delivery time had the strongest impact on the proportion of lymphocytes receiving ≥0.5Gy: best CL-sparing was achieved with 10MV FFF 3DCRT followed by VMAT. However, the clinical parameters metastasis location and in particular metastasis volume had the strongest impact on the immunosuppressive effects of radiotherapy. We therefore believe that these results will be relevant in the setting of combined radio-immunotherapy with potential implications for immune-optimized treatment planning in the future.

## Additional files

Additional file 1: Segment volume. Volume and blood flow per segment. Calculated absolute and relative volume and blood flow of the individual liver segments. (PDF 129 kb)

Additional file 2: Segment distance. Distance to geometric center and mean hepatic transition time per segment. 2D Distance from arterial blood supply/venous drainage to geometric center of individual segments. 3D Pythagorean distance calculation for estimation of mean hepatic transition time per segment (in seconds). (PDF 148 kb)

Additional file 3: DVH convulution algorithm. DVH convolution algorithm. For every treatment fraction, the current Blood DVH is multiplied by a new convolution DVH consisting of individual liver segments & the blood fraction outside the liver. As a result, a new Blood DVH is generated. (PDF 726 kb)

Additional file 4: Fractionation effect on CL (3D CRT). Fractionation effect on CL (3DCRT). There is no significant fractionation effect on circulating lymphocytes with 3DCRT for the apical tumor location. (PDF 26 kb)

## Abbreviations

BOT: Beam-on time
CL: Circulating lymphocytes
CL0.5: Proportion of circulating lymphocytes receiving ≥0.5Gy
CRT: Conventional radiotherapy
MLD: Mean liver dose
SBRT: Stereotactic body radiotherapy
TLC: Total lymphocyte count
VMAT: Volumetric arc therapy
