# Supplementary material for: Modelling the immunosuppressive effect of liver SBRT by simulating the dose to circulating lymphocytes: an in-silico planning study
Source: Radiat Oncol. 2018 Jan 22;13:10. doi: 10.1186/s13014-018-0952-y (PMC5778751; doi:10.1186/s13014-018-0952-y)
Supplement: Supplementary file 1 — Segment volume. Volume and blood flow per segment. Calculated absolute and relative volume and blood flow of the individual liver segments. (PDF 129 kb) [file 13014_2018_952_MOESM1_ESM.pdf]

Additional file 1: Absolute and relative volume and blood flow of individual segments

Total blood volume (TBV): 5000 ml
Blood flow velocity: 10 mm/s

Volume and blood flow per segment				
**Structure**	**Absolute Volume (cm3)**	**Relative Volume (%)**	**Absolute blood flow (ml/min)**	**Relative blood flow (%)**
**Liver**	1194.8	100.0%	1475.00	29.5%
**Segment I**	24	2.0%	29.63	0.6%
**Segment II**	141.8	11.9%	175.05	3.5%
**Segment III**	69.9	5.9%	86.29	1.7%
**Segment IV**	74.5	6.2%	91.97	1.8%
**Segment V**	232.4	19.5%	286.90	5.7%
**Segment VI**	126	10.5%	155.55	3.1%
**Segment VII**	265.7	22.2%	328.01	6.6%
**Segment VIII**	232.7	19.5%	287.27	5.7%
