# Supplementary material for: Modelling the immunosuppressive effect of liver SBRT by simulating the dose to circulating lymphocytes: an in-silico planning study
Source: Radiat Oncol. 2018 Jan 22;13:10. doi: 10.1186/s13014-018-0952-y (PMC5778751; doi:10.1186/s13014-018-0952-y)
Supplement: Supplementary file 2 — Segment distance. Distance to geometric center and mean hepatic transition time per segment. 2D Distance from arterial blood supply/venous drainage to geometric center of individual segments. 3D Pythagorean distance calculation for estimation of mean hepatic transition time per segment (in seconds). (PDF 148 kb) [file 13014_2018_952_MOESM2_ESM.pdf]

Additional file 2: 2D Distance from arterial blood supply / venous drainage to geometric center of individual segments. 3D Pythagorean distance calculation for estimation of mean hepatic transition time per segment (in seconds).

Distance to geometric center and mean hepatic transition time per segment

Segment	Axial distance (cm)	Coronal distance (cm)	Sagittal distance (cm)	Axial square (cm2)	Coronal square (cm2)	Sagittal square (cm2)	Sum of squares (cm2)	Square root (cm)	Square root x 2 (cm)	Mean hepatic transition time (s)
I	1.74	2.4	1.56	3.03	5.76	2.43	11.22	3.35	6.70	7
II	7.13	7.55	4.42	50.84	57.00	19.54	127.38	11.29	22.57	23
III	4.9	2.97	4	24.01	8.82	16.00	48.83	6.99	13.98	14
IV	4.31	2.82	4.49	18.58	7.95	20.16	46.69	6.83	13.67	14
V	5.53	5.85	2.3	30.58	34.22	5.29	70.09	8.37	16.74	17
VI	7.21	4.81	5.6	51.98	23.14	31.36	106.48	10.32	20.64	21
VII	6.44	4.91	6.55	41.47	24.11	42.90	108.48	10.42	20.83	21
VIII	5.12	5.98	2.99	26.21	35.76	8.94	70.91	8.42	16.84	17

2D-Distance to geometric segment center

Single 3D-distance to geometric segment center (cm) in cm

Double 3D-distance to geometric segment center (cm) in cm
