# Supplementary material for: Modelling the immunosuppressive effect of liver SBRT by simulating the dose to circulating lymphocytes: an in-silico planning study
Source: Radiat Oncol. 2018 Jan 22;13:10. doi: 10.1186/s13014-018-0952-y (PMC5778751; doi:10.1186/s13014-018-0952-y)
Supplement: Supplementary file 3 — DVH convulution algorithm. DVH convolution algorithm. For every treatment fraction, the current Blood DVH is multiplied by a new convolution DVH consisting of individual liver segments & the blood fraction outside the liver. As a result, a new Blood DVH is generated. (PDF 726 kb) [file 13014_2018_952_MOESM3_ESM.pdf]

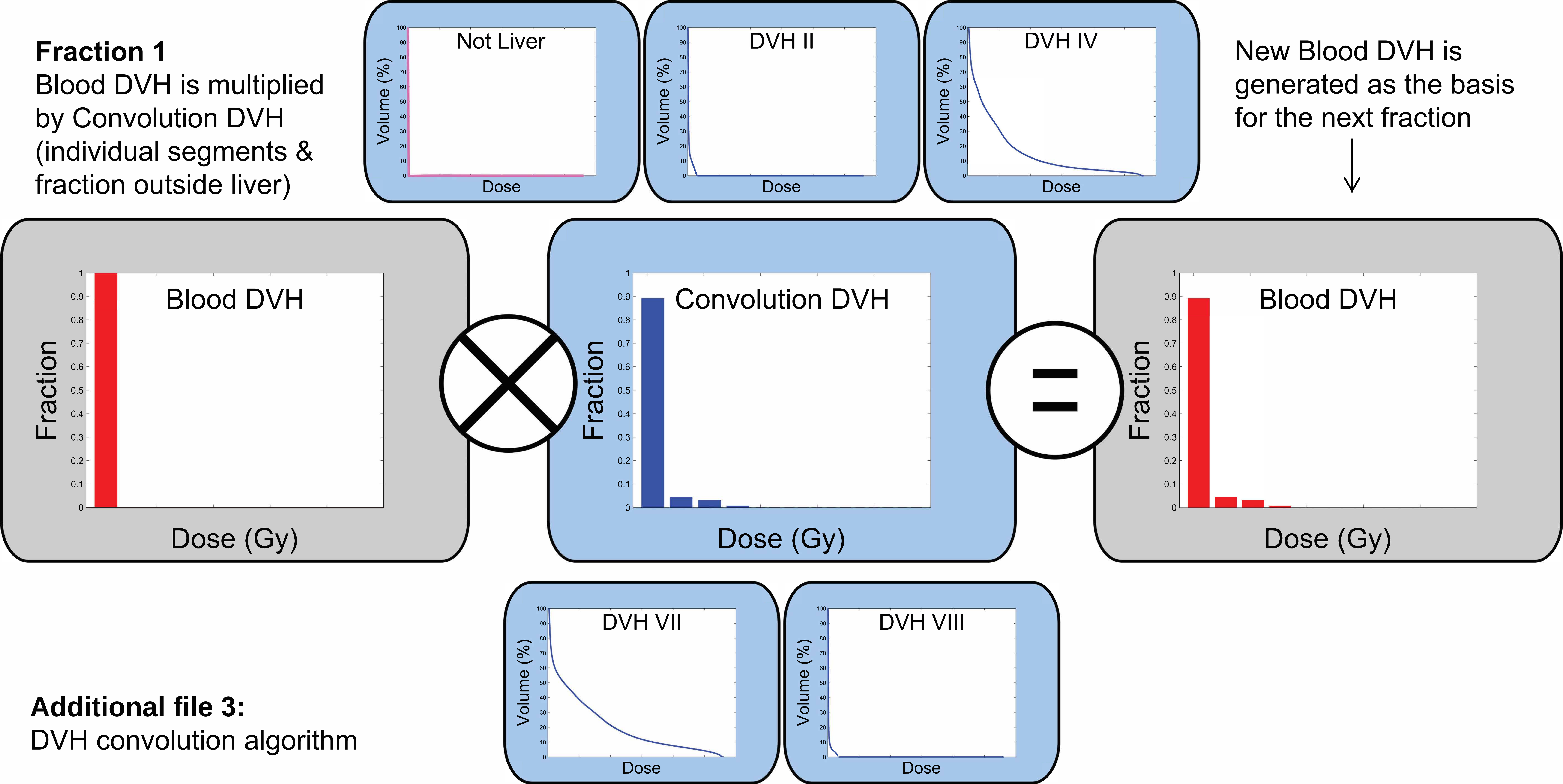

Additional file 3:
DVH convolution algorithm

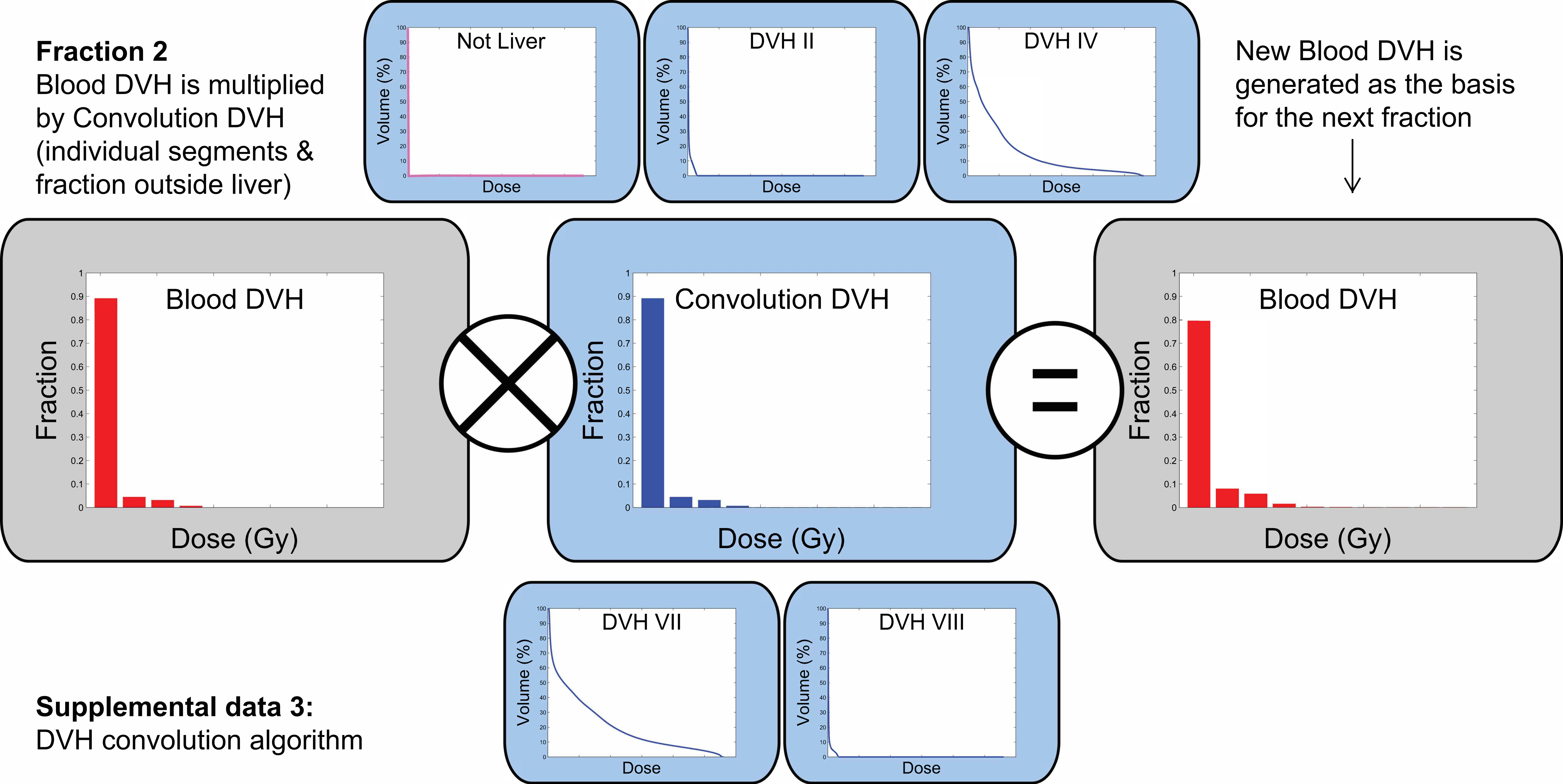

Supplemental data 3:
DVH convolution algorithm

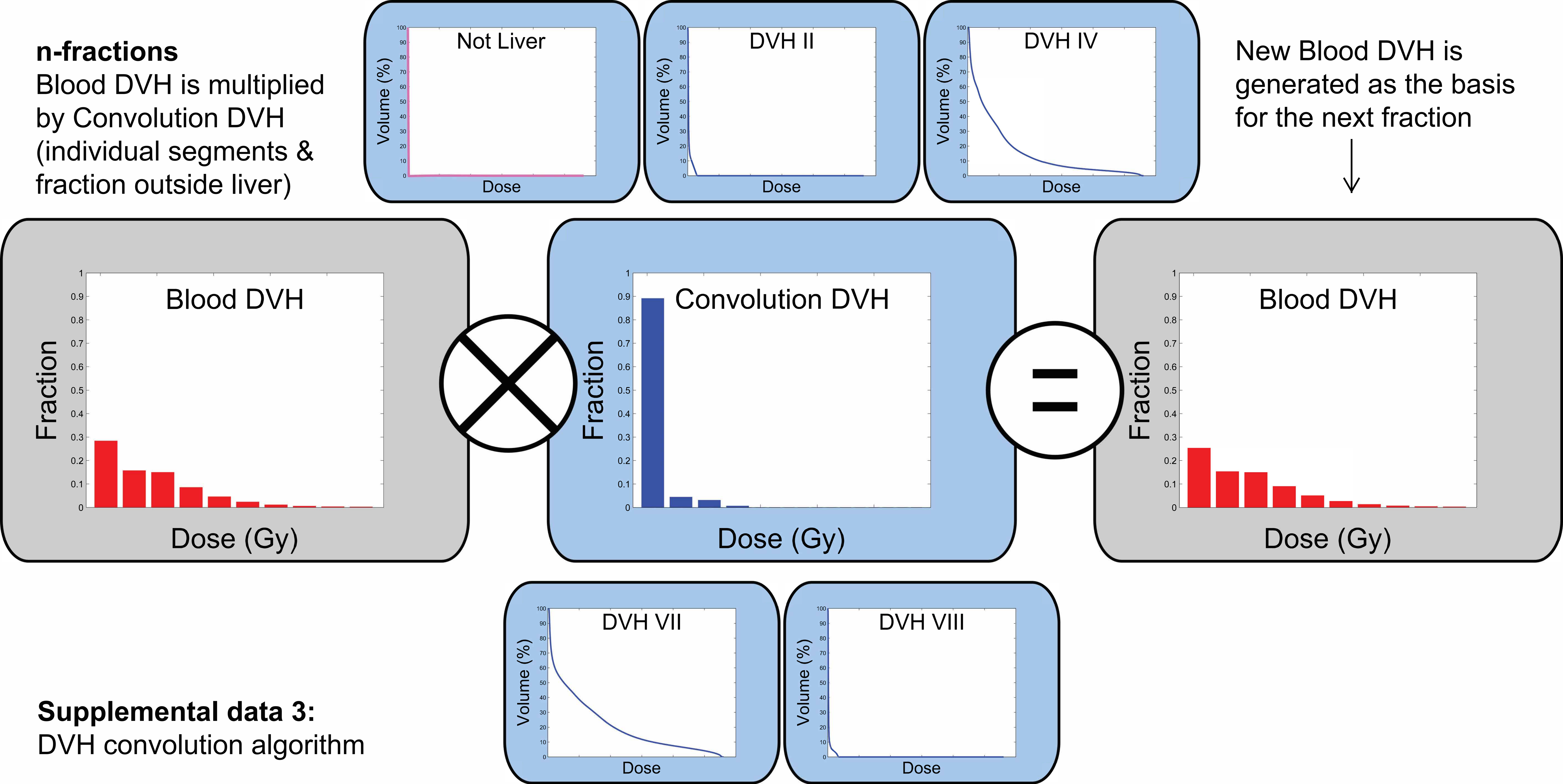

Supplemental data 3:
DVH convolution algorithm
