# Supplementary figures and images for: Modelling the immunosuppressive effect of liver SBRT by simulating the dose to circulating lymphocytes: an in-silico planning study
Source: Radiat Oncol. 2018 Jan 22;13:10. doi: 10.1186/s13014-018-0952-y (PMC5778751; doi:10.1186/s13014-018-0952-y)

# Fractionation effect on CL (3DCRT) for the apical tumor location

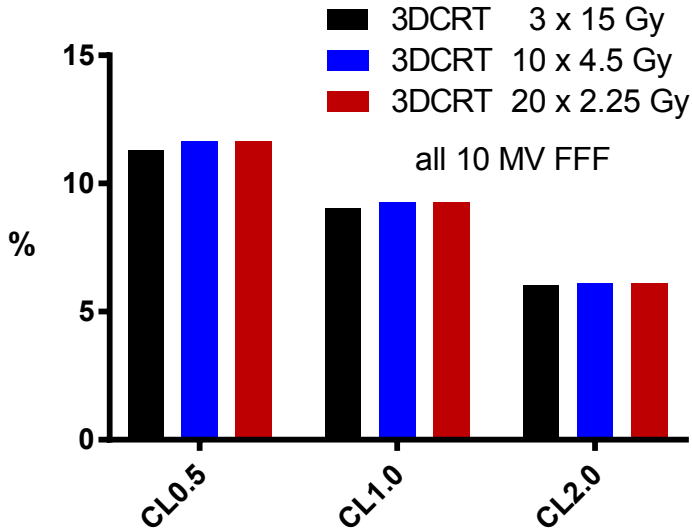

Supplement: Supplementary file 4 — Fractionation effect on CL (3D CRT). Fractionation effect on CL (3DCRT). There is no significant fractionation effect on circulating lymphocytes with 3DCRT for the apical tumor location. (PDF 26 kb) [file 13014_2018_952_MOESM4_ESM.pdf]
